# Supplementary material for: Experience and Resilience of Japanese Public Health Nurses during the COVID-19 Pandemic and Their Impact on Burnout
Source: Healthcare (Basel). 2023 Apr 13;11(8):1114. doi: 10.3390/healthcare11081114 (PMC10137901; doi:10.3390/healthcare11081114)
Supplement: Supplementary file 1 [file healthcare-11-01114-s001.zip › healthcare-2256523-supplementary.pdf]

Supplementary file
Questionnaire

Demographic variables

| | |
|---|---|
| 1. Sex | Male/ Female/ Other |
| 2. Age | |
| 3. Career as PHN | |
| 4. Type of municipality | Prefecture/ Core City/ Special ward in Tokyo metropolis/ Other |
| 5. occupational position | Entry-level/ Mid-level/ Administrative positions |
| 6. Commute time for one-way | less than 30 min/ 30 min to 1 hour/ 1hour to 1.5hour/ more than 1.5hour |
| 7. "Do you have children below middle school?" | Yes, No |
| 8. "Do you live with family member in need of nursing care?" | Yes, No |
| 9. maximum overtime hours per month, and when | 1st wave (January to May 2020)/ 2nd wave (June to October 2020)/ 3rd wave (November 2020 to March 2021)/ 4th wave (April to June 2021)/5th wave (July to September 2021)/ from 6th wave onwards (from January 2022) |

When was the most distressful period?

1. 1st wave (January to May 2020)
2. 2nd wave (June to October 2020)
3. 3rd wave (November 2020 to March 2021)
4. 4th wave (April to June 2021)
5. 5th wave (July to September 2021)
6. from 6th wave onwards (from January 2022)

Why did you choose the period in the previous question? (Free description)

Did you experience the following situation during the COVID-19 pandemic?
(1 = Strongly disagree, 2 = Disagree, 3 = Neutral, 4 = Agree, 5 = Strongly agree)

1. Difficulty in coordinating medical care for patients owing to lack of medical resources
2. Without the concept of "full beds," duties of dealing with patients are endless

Supplementary file
Questionnaire

3.Complaints and verbal abuse from community residents about the PHC's response
4.Unreasonable demands or long hours of complaints from COVID-19-positive patients
5. Difficulty in providing smooth health instructions to foreign community residents
6. Difficulty in gaining understanding of requests for cooperation in preventing the spread of infection from close contacts without a legal basis
7. Being occupied with medical management for individuals, incapable of performing important duties for preventing infection spread
8. Inappropriate staff allocation from outside and within PHCs
9. Lack of uniformity within PHC (e.g., decisions on dealing with community residents, awareness of tasks, overall flow)
10. Overtime/holiday work changes the life pattern

How do you agree to following items to yourself and your workplace environment during the COVID-19 pandemic?
(1 = Strongly disagree, 2 = Disagree, 3 = Neutral, 4 = Agree, 5 = Strongly agree)
1. Managers have leadership skills (e.g., quick decision-making, clear instructions, leading the response to difficult cases)
2. There is a PHN who assists and supports the PHN manager
3. Managers create a comfortable working environment. (e.g., culture of openness, careful attention to individual health)
4. In responding to emergencies, the arrangement and management of work within the PHC organization are reviewed
5. There is a situation-specific response by the central government (e.g., establishing new departments, outsourcing of duties, dispatching staff)
6. Support staff from outside of PHCs is kept fixed for the long term and allows accurate response to tasks
7. In-office systems are constantly made and improved as necessary (e.g., developing tools for sharing information)
8. A system for receiving support staff into PHCs is well prepared and coordinated as appropriate
9. Feeling free to share conflicts and personal feelings within the organization
10. There is a sense of awareness and common understanding of the disaster response to be undertaken by all staff in PHCs
11. A shift in working styles in anticipation of long-term pandemics (e.g., introducing an overtime rotation system)
